# Evaluation of efficacy and safety of traditional Chinese medicine in the treatment of hip synovitis in children: A protocol for systematic review and meta-analysis

**DOI:** 10.1097/MD.0000000000027960

**Published:** 2021-11-24

**Authors:** Junming Chen, Peilin He, Qianhua Liu, Ning Liu, Youwen Liu, Chen Yue

**Affiliations:** a Chengdu University of Traditional Chinese Medicine, Chendu, China; b Key Laboratory of Orthopedics & Traumatology of Traditional Chinese Medicine and Rehabilitation Ministry of Education, Fujian University of TCM, Fuzhou, China; c Department of Orthopedic Surgery, Luoyang Orthopedic Hospital of Henan Province, Orthopedic Hospital of Henan Province, Luoyang, China; d Fujian University of Traditional Chinese Medicine, Fuzhou, China.

**Keywords:** efficacy and safety, hip synovitis in children, protocol, systematic review, traditional Chinese medicine

## Abstract

**Background:**

Hip synovitis is a common hip disorder in children and a frequent cause of hip or groin pain in children. Its onset is rapid and poses a threat to patient health. Conventional treatment methods have suboptimal efficacy and large side effects. Clinical study surface, the therapeutic effect of traditional Chinese medicine (TCM) on hip synovitis in children is obvious. Therefore, we aimed to systematically review the efficacy and safety of TCM on hip synovitis in children.

**Methods:**

We will search databases including PubMed, Web of Science, EMBASE database, Cochrane Library, MEDLINE, Wanfang Data, Chinese biomedical literature database, China National Knowledge Infrastructure, Chinese science and technology journals database, and World Health Organization International Clinical Trials Registry Platform (since the databases were established). We also searched secondary resources, including the reference lists of studies. Included articles were carefully screened and reviewed by 2 researchers. Statistical analysis was performed using Review Manager 5.3 software.

**Results:**

This study will comprehensively evaluate the efficacy and safety of TCM for the treatment of hip synovitis in children.

**Conclusion:**

This systematic review explores the efficacy and safety of TCM for the treatment of hip synovitis in children and provides an update on its clinical use.

## 1 Introduction

Synovitis of the hip in children is a non-specific inflammatory change of the hip, with transient acute pain, swelling, and effusion as the main clinical manifestations. With an incidence of approximately 3:1 in men and women, it is self-limiting and is the most common cause of hip pain in children aged 3 to 8 years.^[1,2]^ Its etiology has not been fully determined until now and is generally considered as temporary hip pain and dysfunction. Caused by bacterial or viral infection, hip joint damage, excessive fatigue, allergy, and so on. The disorder is usually acute, with the child complaining of hip pain, limping, or refusal to walk.^[3–5]^ If the disease is not correctly diagnosed at an early stage, without active treatment, and the course of the disease is prolonged, it not only affects hip function but may even evolve to avascular necrosis of the femoral head, triggering developmental disorders of the hip in children.^[6,7]^ At present, the clinical drug treatment of the disease is mainly using oral non-steroidal anti-inflammatory drugs, intravenous antibiotics, anti-viral drugs, etc, all have adverse drug reactions and safety problems; Surgical treatment of this disease has certain trauma and risks.^[8–10]^ Therefore, finding the optimal treatment for synovitis of the hip in children is a difficult task for clinicians.

In recent years, traditional Chinese medicine (TCM) has played an important role in the prevention and treatment of arthritis, and there are more and more clinical studies on TCM treating hip synovitis in children, and the TCM can directly act on the lesion site, accelerate the disappearance of inflammation, accelerate blood circulation, and promote the improvement of clinical symptoms.^[11–14]^ Currently, there is still a lack of relevant systematic reviews in clinical practice, and the aim of this protocol is to evaluate the efficacy and safety of TCM for the treatment of hip synovitis in children.

## 2 Methods

### 2.1 Study registration

Our systematic review and meta-analysis protocol registration number was: INPLASY2021100087. We have full registration information available at inpasy.com. Our report will follow the PRISMA recommendations.^[15]^

### 2.2 Ethics and dissemination

Since all information and data for this systematic review protocol were extracted from the network and did not involve recruiting patients, ethical approval was not required for this systematic review protocol.

### 2.3 Eligibility criteria for included studies

#### 2.3.1 Types of studies

Only randomized controlled trials will be used to evaluate the efficacy and safety of TCM in the treatment of hip synovitis in children; case reports, conferences, animal studies, and non-randomized studies will be excluded, with no restrictions on publication type or language.

#### 2.3.2 Types of participants

Participants in this study had to meet the diagnostic criteria for synovitis of the hip in children, which is diagnosed through 3 areas: physical examination, imaging, and laboratory tests. Gender, age, and treatment course were not restricted.

#### 2.3.3 Types of interventions

Patients in the experimental group should receive conventional TCM treatment, and subgroup analysis was performed to analyze different TCMs with different treatment durations, while patients in the control group received conventional treatments: oral non-steroidal anti-inflammatory drugs, static antibiotics, antiviral drugs, skin traction, surgical treatment, etc.

#### 2.3.4 Types of outcome measures

##### 2.3.4.1 Primary outcomes

The primary outcomes were Harris hip score and hip ultrasound level.

##### 2.3.4.2 Secondary outcomes

The secondary outcome was pain visual analogue scale score.

### 2.4 Database search strategy

Use computer search and manual search for relevant articles, the retrieved databases included PubMed, Web of Science, EMBASE database, Cochrane Library, MEDLINE, Wanfang Data, Chinese biomedical literature database, China National Knowledge Infrastructure, Chinese science and technology journals database, and World Health Organization International Clinical Trials Registry Platform. All the retrieved literature was established from the database to today. The different retrieval methods were adapted according to different database characteristics. We briefly described the search process of PubMed (Table 1).^[16]^

**Table 1:** Search strategy for PubMed.

Number	Search terms
#1	traditional chinese medicine fumigation[MeSH Terms]
#2	herbal fumigation[Title/Abstract]
#3	chinese herbal fumigation[Title/Abstract]
#4	chinese medicine fumigation[Title/Abstract]
#5	chinese herb fumigation[Title/Abstract]
#6	traditional herbal fumigation[Title/Abstract]
#7	traditional chinese medicine steaming[Title/Abstract]
#8	or/#1-#7
#9	hip synovitis children[All Fields]
#10	hip synovitis[All Fields]
#11	hip joint synovitis[All Fields]
#12	coxa synovitis[All Fields]
#13	or/#9-#12
#14	randomized controlled trial [Title/Abstract]
#15	controlled clinical trial [Title/Abstract]
#16	randomized [Title/Abstract]
#17	randomised [Title/Abstract]
#18	placebo [Title/Abstract]
#19	randomly [Title/Abstract]
#20	trial [Title/Abstract]
#21	groups [Title/Abstract]
#22	or/#14-#21
#23	#8and#13and#22

### 2.5 Study selection and data extraction

Titles and abstracts of the obtained studies were independently reviewed by 2 investigators, and studies that did not meet the inclusion criteria were excluded. Investigators read the full texts to identify eligible studies. If an investigator disputed the findings, then the assistance of a third investigator was enlisted.^[17]^

Data extraction items included: first author, publication year, gender, age, country, intervention method, number of participants, disease duration, treatment course, and outcome.

### 2.6 Assessment of risk of bias

Two investigators independently assessed the methodological quality of each included study according to the Cochrane Handbook for Systematic Reviews 5.3 (https://www.cochrane.org/) as the recommended assessment tool. Any disagreement between investigators was resolved by discussion.

### 2.7 Statistical analysis

We adopted Revman 5.3 (Cochrane Collaboration) software for all statistical analyses. The chi-square test and *I*^*2*^ statistic were used to measure heterogeneity between studies. If any substantial heterogeneity was found (*P* < .05 and *I*^*2*^ > 50%), then we used random-effects model statistics; otherwise, we used the fixed effects model for statistics. All data analyses were performed with 95% confidence intervals. Continuous data were analyzed as the mean difference or the normalized mean difference, whereas dichotomous data were considered as the relative risk. In cases of *P* < .05, the difference was statistically significant. Lastly, we used sensitivity analysis to assess the stability of the results. If necessary, funnel plots and egger tests were created and checked to ensure that there was no bias in the publication.

## 3 Discussion

Synovitis of the hip joint in children is an acute orthopedic disease with a short disease course, benign, and can be completely cured, and the effusion can be completely absorbed. Clinically, since children do not cooperate with the clinical examination of doctors, it will lead to the clinical examination of doctors is not careful enough, easily missed and misdiagnosed, resulting in serious consequences.^[1,18–20]^

TCM has extensive experience in treating synovitis of the hip joint in children, and the efficacy is significant. This is the first meta-analysis of the efficacy and safety of TCM for the treatment of hip synovitis in children, and we hope that this will facilitate the development and use of TCM to better treat patients.

## Author contributions

**Conceptualization:** Junming Chen, Chen Yue.

**Data curation:** Junming Chen, Peilin He, Qianhua Liu, Ning Liu, Youwen Liu, Chen Yue.

**Formal analysis:** Junming Chen, Peilin He, Chen Yue.

**Methodology:** Junming Chen, Peilin He, Chen Yue.

**Supervision:** Chen Yue.

**Writing – original draft:** Junming Chen, Peilin He, Qianhua Liu, Chen Yue.

**Writing – review & editing:** Junming Chen, Peilin He, Qianhua Liu, Ning Liu, Youwen Liu, Chen Yue.
